# Hydrogen Peroxide in Inflammation: Messenger, Guide, and Assassin

**DOI:** 10.1155/2012/541471

**Published:** 2012-06-12

**Authors:** C. Wittmann, P. Chockley, S. K. Singh, L. Pase, G. J. Lieschke, C. Grabher

**Affiliations:** ^1^Institute of Toxicology and Genetics, Karlsruhe Institute of Technology (KIT), 76133 Karlsruhe, Germany; ^2^Cell Cycle and Cancer Genetics Laboratory, Peter MacCallum Cancer Centre, East Melbourne, VIC 3002, Australia; ^3^Australian Regenerative Medicine Institute, Monash University, Clayton, VIC 3800, Australia

## Abstract

Starting as a model for developmental genetics, embryology, and organogenesis, the zebrafish has become increasingly popular as a model organism for numerous areas of biology and biomedicine over the last decades. Within haematology, this includes studies on blood cell development and function and the intricate regulatory mechanisms within vertebrate immunity. Here, we review recent studies on the immediate mechanisms mounting an inflammatory response by *in vivo* analyses using the zebrafish. These recently revealed novel roles of the reactive oxygen species hydrogen peroxide that have changed our view on the initiation of a granulocytic inflammatory response.

## 1. Introduction

The innate immune system comprises the cells and mechanisms that defend the host from infection by other organisms or damage to tissue integrity, in a nonspecific manner. This means that the cells of the innate system recognise and respond to pathogens and trauma in a generic way, but unlike the adaptive immune system, it does not confer long-lasting or protective immunity to the host. The innate immune system provides an immediate defence. A typical vertebrate immune response depends on the orchestrated motility and activity of various haematopoietic compartments and their interactions that ultimately control the magnitude of the response [1–3]. Inflammation is one of the first responses of the immune system to infection or irritation. Stimulated by factors released from injured cells, it serves to establish a physical barrier against the spread of infection. This further promotes healing of any damaged tissue following the clearance of pathogens or cell debris. Molecules produced during inflammation sensitise pain receptors, cause localised vasodilatation of blood vessels, and attract phagocytes, especially neutrophils and macrophages, which then trigger other parts of the immune system.

Failure to initiate a response allows uncontrolled proliferation of invading microorganisms and severe tissue damage that may become fatal. Failure to resolve an immune response can also cause severe tissue damage, due to persistent degranulation, and may lead to chronic inflammation, which ceases to be beneficial to the host. Overall, inflammation is now recognised as a central feature of prevalent pathologies, such as atherosclerosis, cancer, asthma, thyroiditis, inflammatory bowel disease, autoimmune disease, as well as Alzheimer's and Parkinson's disease [4–6]. Hence, the regulation of an inflammatory response is an active field of research. New players or novel functions of old players continue to be identified and we are only beginning to understand their specific function at the corresponding level during inflammation. Hydrogen peroxide is an example of a molecule with a long known function for pathogen clearance in inflammation. Here, we discuss how recent work using the zebrafish model has revealed a pivotal role of hydrogen peroxide in mounting an inflammatory response.

## 2. Cellular Lifecycle of Hydrogen Peroxide

Hydrogen peroxide belongs to a group of chemically reactive molecules known as reactive oxygen species (ROS) that arise through oxidative metabolism. ROS comprise oxygen derived small molecules such as the oxygen radicals: superoxide, hydroxyl, peroxyl, and alkoxyl; or the nonradicals: hypochlorous acid, ozone, singlet oxygen, and the current topic in focus, hydrogen peroxide [7]. ROS generation can either occur as a by-product of cellular metabolism (e.g., in mitochondria through autoxidation of respiratory chain components) or it can be created by enzymes with the primary function of ROS generation [8]. Enzymes capable of rapidly increasing local H_2_O_2_ levels include the family of NADPH oxidases [7] and other oxidases such as xanthine oxidase [9] and 5-lipoxygenase [10]. The mammalian NADPH oxidase family encompasses 7 members, which are NOX1-5 and DUOX1-2. To date, a single isoform of *duox* and four *nox* genes (*nox1, 2, 4, 5*) have been identified in the zebrafish genome [11]. Each member is capable of converting NADPH to NADP^+^ and then transporting the freed electrons across membranes. DUOX enzymes are capable of direct hydrogen peroxide production, while NOXes1-5 produce superoxide, which is rapidly converted to H_2_O_2_ by a separate superoxide dismutase or occurs spontaneously [12]. H_2_O_2_ may subsequently be utilised by peroxidase, such as thyroperoxidase, to produce thyroid hormones or myeloperoxidase and lactoperoxidase to generate more potent ROS. However, if not consumed, high concentrations of H_2_O_2_ may result in DNA damage and modifications of proteins, lipids and other molecules [13]. Thus, to avoid H_2_O_2_-mediated deleterious effects, excess H_2_O_2_ is usually rapidly catalysed or reduced by various antioxidant enzymes: such as glutathione peroxidase and catalase [14].

## 3. Functional Activities of H_2_O_2_

H_2_O_2_ is also involved in many regulatory cellular events including the activation of transcription factors, cell proliferation, and apoptosis [8]. H_2_O_2_ produced from the mitochondrial electron transport chain has been shown to play a role in haematopoietic cell differentiation and cell division in flies [15, 16]. NADPH oxidase generated H_2_O_2 _can affect cardiac differentiation [17], vascularisation [18], and angiogenesis [19]. In targeting cysteine and methionine residues of protein kinases and phosphatases, H_2_O_2_ is capable of modulating a number of principal signalling cascades including ERK, JNK, p38, MAPK, and PI3K/Akt [20, 21].

### 3.1. Inflammation-Related Functions

#### 3.1.1. Respiratory Burst

The classical physiological role attributed to H_2_O_2_ is its capability to induce bacterial killing [12]. NOX2 is the enzyme responsible for phagocyte respiratory burst responses and is expressed in neutrophils, eosinophils, monocytes/macrophages, as well as nonphagocytic cells such as fibroblasts, cardiomyocytes, haematopoietic stem cells, and endothelial cells [7]. Under resting conditions neutrophil NOX2 resides in secondary granules, which upon activation of neutrophils fuse with phagosomal as well as plasma membranes [22].

The NADPH-oxidase-mediated respiratory burst response of neutrophils generates two superoxide anions by transporting two electrons from one NADPH across the membrane to the extracellular or intra-phagosomal space. Superoxide is further converted into hydrogen peroxide either through spontaneous dismutation, which involves the consumption of two protons, or facilitated by the catalytic activity of superoxide dismutase. Hydrogen peroxide alone and in conjunction with the amplification activity of myeloperoxidase (MPO) is responsible for bacterial killing [23, 24]. MPO, which is abundantly present in phagocyte granules, catalyses the conversion of halides and pseudohalides such as Cl^−^, I^−^, Br^−^, and SCN^−^ to form hypohalous acids or pseudohypohalous acids. HOCl, however, is the primary MPO product in neutrophils responsible for bacterial killing.

#### 3.1.2. Hydrogen Peroxide Mounting an Inflammatory Response

Recent advances accomplished by utilising the model organism zebrafish greatly expanded our view of H_2_O_2_ mediated cellular activities. The optical transparency of zebrafish larvae offers the unique advantage of real-time monitoring an immune response in a whole animal context. This is in contrast to *in vitro* studies and/or end-point analyses of stained tissues. Additionally, a recently developed genetically encoded H_2_O_2_ sensor provided an elegant solution for investigating the role of hydrogen peroxide dynamics during an immune response *in vivo* [25].

The previous view on the critical mechanisms in immediate inflammation focused on the activity of damage-associated molecular patterns (DAMPs) and pathogen-associated molecular patterns (PAMPs). Tissue damage results in the release of intracellular DAMPs usually hidden from the immune system (i.e., ATP, uric acid, lipids, DNA, nuclear proteins) or extracellular DAMPs released through degradation of extracellular matrix upon tissue injury (i.e., hyaluronan, byglycan, heparan sulfate). The receiving cell senses these signals through 5 different types of pattern recognition receptors (PRRs). Activation of these receptors in turn activates downstream NFkB, MAPK, or type I interferon-signalling pathways that are important for inflammatory and antimicrobial responses. The significance of DAMPs, PAMPs, and PRRs is comprehensively reviewed elsewhere [26, 27]. However, the mechanisms for immediate immune cell recruitment were not well defined.

Recently, Niethammer et al. described for the first time that wounded epithelium of zebrafish larvae produces a tissue-scale gradient of H_2_O_2_ mediating leukocyte recruitment [28]. This finding was in contrast to the prevalent view that leukocytes undergoing an oxidative burst response were the only source of H_2_O_2_ at a site of trauma or infection [29]. The authors employed the genetically encoded ratiometric HyPer sensor to visualise H_2_O_2_ *in vivo* and in real time. HyPer consists of the bacterial H_2_O_2_-sensitive transcription factor, OxyR, fused to a circularly permutated yellow fluorescent protein (YFP). Cysteine oxidation of OxyR induces a conformational change in the YFP that increases emission excited at 500 nm and decreases emission excited at 420 nm. This change is rapidly reversible within the reducing cytoplasmic environment, which allows dynamic monitoring of the intracellular hydrogen peroxide concentration [30].

Tailfin transection on zebrafish larvae induced a rapid increase in H_2_O_2_ levels extending approximately 100–200 *μ*m from the wound margin as a decreasing concentration gradient. Furthermore, gradient formation preceded leukocyte arrival at the scene and H_2_O_2_ levels started to decrease again with accumulation of immune cells. Generation of the gradient as well as leukocyte recruitment was dependent on the activity of Duox in epithelial cells. Both, genetic knockdown of Duox and chemical inhibition of oxidase activity abolished gradient formation and significantly decreased absolute numbers of leukocytes at the wound margin, without affecting general cellular motility. These findings were corroborated by a study in drosophila focusing on prioritising competing signals by migrating macrophages [31] emphasising the crucial role of the tissue scale gradient of H_2_O_2_ for leukocyte attraction.

A study, also using zebrafish larvae, demonstrated that newly oncogene-transformed cells and their neighbours attracted leukocytes through H_2_O_2_ signalling. Utilising the H_2_O_2_-indicating dye, acetyl-pentafluorobenzene sulphonyl fluorescein, and 5,5-dimethyl-l-pyrroline N-oxide (DMPO) that reports a history of ROS exposure, it was shown that H_2_O_2_ was stochastically and momentarily produced around V12RAS expressing cells in the epidermis. Like wounded epithelial cells, transformed cells generated H_2_O_2_ in a Duox dependent manner, highlighting parallels between oncogene-transformed cells and mechanical induced injury initiation of the host inflammatory response [32].

#### 3.1.3. Hydrogen Peroxide as a Signalling Molecule in Inflammation

Functional roles of H_2_O_2_ during inflammation have been observed previously. Mechanistically, hydrogen peroxide can modulate protein function by reversible chemical modification of protein thiols, which can result in conformational changes affecting DNA binding, enzymatic activity, multimerisation, or protein complex formation. For example, the NFkB/Rel family, key regulatory molecules in the transcription of many genes involved in inflammation, is a well-known redox-sensitive transcription factor family [33]. H_2_O_2_-induced activation of NFkB, which includes tyrosine phosphorylation of IkB and activation of IKK by H_2_O_2_ has been reported [34, 35]. Moreover, H_2_O_2_ can activate the release of high mobility group 1 protein from macrophages resulting in amplification of proinflammatory stimuli [36] or modulate leukocyte adhesion molecule expression and leukocyte endothelial adhesion [29]. VCAM-1, an endothelial scaffold on which leukocytes migrate, can activate signals in endothelial cells required for VCAM-1-dependent leukocyte migration. Leukocyte binding to VCAM-1 stimulates NOX2 in endothelial cells, resulting in the generation of H_2_O_2_, which locally activates matrix metalloproteinases (MMPs). These MMPs in turn degrade matrix and endothelial cell surface receptors in cell junctions facilitating leukocyte transendothelial migration [37, 38].

These examples show how H_2_O_2_ can act as an intracellular or local signalling molecule but long-distance intercellular mechanisms of H_2_O_2_-mediated leukocyte recruitment were less well defined.

The open question of how leukocytes may receive the signal to initiate directional migration was recently addressed in another elegant study using the zebrafish model by Yoo et al. [39]. They have identified the SRC family kinase (SFK) Lyn as a redox sensor in neutrophils that detects hydrogen peroxide emanating from wounds and guiding their migration. Yoo and colleagues were able to provide direct evidence for punctate SFK activation at the leading edge of neutrophils in response to wounding. Through the knockdown of Duox, which is responsible for H_2_O_2_ production at the wound margin, they have explored the role of H_2_O_2_ in SFK activation. Duox knockdown prevented SFK phosphorylation indicating that activation of neutrophil SFKs may be dependent on the presence of hydrogen peroxide levels at wounds. Further evidence suggesting that SFKs can act as a redox sensor was provided by utilisation of SFK inhibitors that resulted in impairment of early neutrophil accumulation, while having no effect on epithelial hydrogen peroxide bursts [39].

Profiling SFK family members in zebrafish myeloid cells identified the Lyn kinase as a promising candidate acting as the redox sensor in neutrophils and macrophages. Morpholino knockdown of Lyn impaired directional migration of neutrophils to a tailfin wound in zebrafish larvae.

Further *in vitro* investigation revealed that hydrogen peroxide directly activates Lyn through the oxidation of Cys466, leading to downstream signalling, for example, Erk activation. This *in vitro* evidence was elegantly confirmed *in vivo* using a combination of genetic knockdown of Lyn and neutrophil-specific transgenic reconstitution of a Cys466 mutant or wild-type Lyn-GFP fusion.

**Figure 1:**
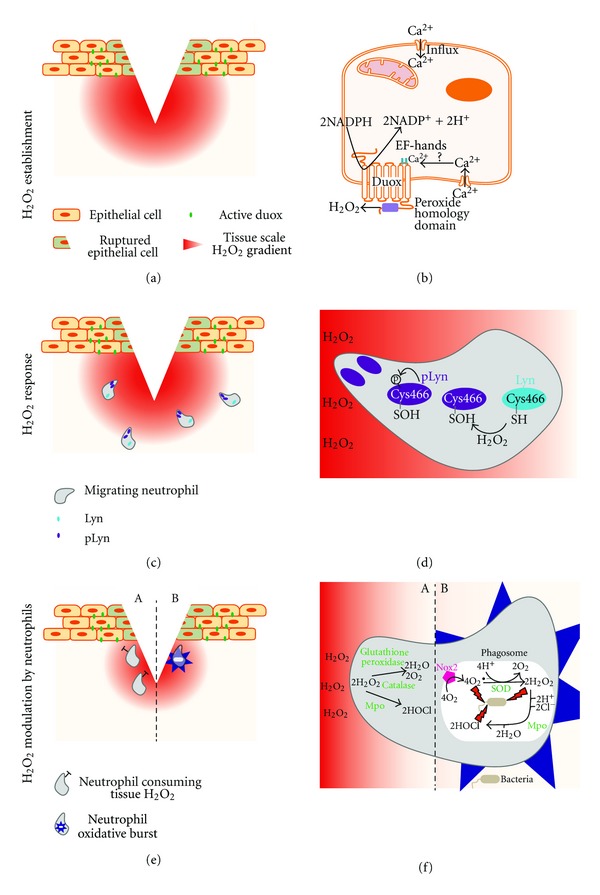
The role of hydrogen peroxide during the inflammatory response. (a) Upon tissue injury/trauma, epithelial cells adjacent to damaged cells activate the NADPH oxidase, Duox. Duox generates and establishes a localised tissue scale gradient of hydrogen peroxide, (b) Potential cellular events that result in Duox activation in epithelial cells. Disruption of epithelial cell membranes by mechanical trauma could lead to an increased influx of calcium in adjacent cells. Calcium binding to the EF-hand domain of Duox (residing in plasma membranes of epithelial cells), may initiate generation of hydrogen peroxide. (c) A tissue scale gradient of hydrogen peroxide acts as the first attraction signal for leukocytes. (d) Neutrophils sense hydrogen peroxide emanating from the wound partly through Lyn, a Src family kinase. Oxidation of Cys466 activates Lyn, resulting in autophosphorylation (pLyn) and punctate appearance of pLyn at the neutrophil leading edge is observed. (e) At the site of injury, neutrophils may alter hydrogen peroxide levels, both by consuming epithelial-derived hydrogen peroxide (A) or by local production of hydrogen peroxide through oxidative bursts (B). (f) Antioxidants, such as glutathione peroxidase and catalase could catalyse the decomposition of hydrogen peroxide into oxygen and water, while myeloperoxidase (Mpo) may consume hydrogen peroxide to produce hypochlorous acid (A). Neutrophils are equipped with multiple mechanisms to kill foreign organisms, one of them being the generation of ROS. Upon activation, phagosomal Nox2 generates superoxide, which is further converted into hydrogen peroxide by superoxide dismutase (SOD). Hydrogen peroxide alone and in conjunction with hypochlorous acid, generated by myeloperoxidase and other ROS exert bactericidal functions (B).

In conclusion, these two sophisticated studies demonstrated a novel role of H_2_O_2_ as mediator of immediate inflammation and revealed aspects of the mechanisms resulting in leukocyte recruitment to a site of trauma (Figure 1). Evidence is accumulating that H_2_O_2_ signalling to phagocytes is a widely conserved mechanism present not only in zebrafish [28, 32, 39] but also flies [31] and mammals [39, 40].

## 4. Outlook

The discovery of a new biological mechanism opens up a new line of research and poses numerous new questions to address. The most obvious being: How is Duox activated in epithelial cells upon wounding and how is the H_2_O_2_ gradient resolved? One hypothesis would place calcium as the immediate injury signal to the wounded cell in order to produce hydrogen peroxide through Duox. Physical disruption of plasma membranes results in an uncontrolled influx of calcium [41]. Giving credence to this hypothesis, evidence exists showing that DUOX activation by calcium regulates H_2_O_2_ generation [42].

In order to avoid excess tissue damage and persistent granulocyte recruitment/retention, the presence of the hydrogen peroxide gradient must be tightly regulated. Regulation could occur on the enzymatic level in terms of H_2_O_2_ production as well as on the molecular level in terms of H_2_O_2_ stability. Oxidase activity results in membrane depolarisation due to the electrogenic properties of the enzymes to the point of NADPH oxidase inhibition. Prolonged H_2_O_2_ production depletes the NADPH pools, which may automatically result in cessation of H_2_O_2_ generation. Alternatively or in addition, neutrophil MPO could be responsible for the decrease in hydrogen peroxide levels upon arrival at the wound [24].

This mechanism suggests new approaches to therapeutically modulate both the onset of the cellular inflammatory response and its resolution, particularly as it involves a small, relatively unstable signalling molecule and is dependent on multiple enzymatic steps amenable to pharmacologic intervention.
